# Precipitating Circumstances of Suicide among Active Duty U.S. Army Personnel Versus U.S. Civilians, 2005–2010

**DOI:** 10.1111/sltb.12111

**Published:** 2014-08-05

**Authors:** Joseph E Logan, Nancy A Skopp, Mark A Reger, Matt Gladden, Derek J Smolenski, C Faye Floyd, Gregory A Gahm

**Affiliations:** Centers for Disease Control and Prevention, National Center for Injury Prevention and ControlAtlanta, GA, USA; Department of Defense, National Center for Telehealth and Technology, Joint Base Lewis-McChordTacoma, WA, USA

## Abstract

To help understand suicide among soldiers, we compared suicide events between active duty U.S. Army versus civilian decedents to identify differences and inform military prevention efforts. We linked 141 Army suicide records from 2005 to 2010 to National Violent Death Reporting System (NVDRS) data. We described the decedents’ military background and compared their precipitators of death captured in NVDRS to those of demographically matched civilian suicide decedents. Both groups commonly had mental health and intimate partner precipitating circumstances, but soldier decedents less commonly disclosed suicide intent.

In recent years, the suicide rate has increased among U.S. soldiers (Bachynski et al., 2012; Kang & Bullman, 2008; Kuehn, 2009). Historically, U.S. service members were less likely to die by suicide compared with civilians (Eaton, Messer, Garvey Wilson, & Hoge, 2006). Helmkamp (1995) reported that the suicide risk among male service members in the 1980s and early 1990s was about half that of civilian males; however, recent reports have stated that the military suicide rate has dramatically increased and has surpassed the civilian rate (Bachynski et al., 2012; Lineberry & O'Connor, 2012). Bachynski et al. (2012) reported that the suicide rate among U.S. Army soldiers nearly doubled between the years of 2003 and 2008.

This rising Army suicide rate has created intense interest in understanding why this rate has increased and how suicide can be prevented among soldiers (Department of Defense, 2012). Research on military suicide has gained momentum in an effort to inform prevention strategies. A few epidemiological studies have now been conducted with military samples. These studies have shown that younger (under 25 years of age) non-Hispanic White males who are enlisted, are divorced, and have lower educational attainment and psychiatric diagnoses are at increased risk of suicide compared with those without such characteristics (Hyman, Ireland, Frost, & Cottrell, 2012; Luxton et al., 2012; Skopp, Trofimovich, Grimes, Oetjen-Gerdes, & Gahm, 2012). Furthermore, firearms have been the most common mechanism used (Logan, Skopp, Karch, Reger, & Gahm, 2012).

Research is still needed to describe military versus civilian suicide incidents to improve understanding of their unique precipitating circumstances, which might inform military suicide prevention efforts (Logan et al., 2012; U.S. Surgeon General & the National Action Alliance for Suicide Prevention, 2012). There are existing data systems in place that could systematically monitor characteristics of suicide incidents in both populations. For example, the Centers for Disease Control and Prevention (CDC) National Violent Death Reporting System (NVDRS) monitors characteristics and precipitating factors of suicide incidents in 18 U.S. states; however, this system does not collect details on military background characteristics among decedents and also does not systematically determine whether decedents were on active duty in the military. Partnering CDC with Department of Defense agencies and linking data sources, such as the Department of Defense Suicide Event Reports (DoDSERs), to NVDRS could help identify a sample of active duty Army suicide decedents in NVDRS. This would allow for a comparison between active duty Army versus civilian suicide decedents with respect to the same NVDRS data elements. The linked DoDSER data would also provide a better description of the decedents’ military characteristics than NVDRS. Attempts to link NVDRS data to DoDSERs have been successful (Logan et al., 2012). In the current study we describe active duty U.S. Army suicide decedents using linked NVDRS-DoD data and compare U.S. Army versus civilian suicide incidents with respect to the precipitating circumstances of death identified from death scene investigation reports captured in NVDRS.

## Methods

### Data Sources

NVDRS provides details on suicide incidents such as decedent characteristics, the mechanisms/weapons involved, and the precipitating circumstances (CDC, 2008). From 2005 to 2010, 18 U.S. states participated in NVDRS; therefore, case inclusion for this study was limited to incidents in those states. From 2005 to 2009, NVDRS included 16 states (Alaska, Colorado, Georgia, Kentucky, Maryland, Massachusetts, New Jersey, New Mexico, North Carolina, Oklahoma, Oregon, Rhode Island, South Carolina, Utah, Virginia, and Wisconsin). In 2010, Ohio and Michigan were added. California also collected data from 2005 to 2008; however, these data were excluded because data collection was not ongoing at the time of the study.

Data sources for NVDRS include law enforcement reports, coroner/medical examiner reports, toxicology reports, and death certificates. All sources are linked by incident into a single repository. States manage data collection through state health departments or subcontracted entities. Data are gathered and coded by trained abstractors (CDC, 2008).

NVDRS details on precipitating circumstances of death come from law enforcement and coroner/medical examiner death scene investigation reports. These reported circumstances were used by investigators to help them determine and justify a manner of death at a violent death scene (e.g., homicide, suicide, etc.). The process that law enforcement and coroner/medical examiner investigators use to gather information on precipitating circumstances of a violent death involve open-ended interviews with family members, friends, and others associated with the decedent as well as witnesses to the death incident to assess what these informants believed led to the death (U.S. Department of Justice, 1999). Investigators corroborate statements, identify commonalities, and compare testimonies to other physical evidence such as a decedent confessional in a suicide note to better understand the motive of the death and before drawing opinions about the manner of death. Law enforcement and coroner/medical examiner investigators offer different perspectives than researchers; however, their incident reports or their conclusions on manner of death are regularly used for research (Logan, Hall, & Karch, 2011; Logan et al., 2008).

The DoD's suicide surveillance program (and the Army's similar program that pre-dated the DoDSER program) provides many details on military suicide decedents that are not captured in NVDRS, such as military background, duty status, combat exposure, and military disciplinary history (Gahm et al., 2012). DoDSER submissions are required for all military suicides within 60 days of notification of a confirmed suicide by the Armed Forces Medical Examiner System. In the Army, DoDSERs are completed by behavioral health providers who are required to submit detailed information through a secure web portal. DoDSER data collection requires medical and mental health records, personnel records, responsible investigative agency records, records related to manner of death, and interviews (e.g., coworkers, supervisors, friends, family members, the responsible investigative agency officer, and other professionals such as physicians, behavioral health clinicians, chaplains, and military police officers), as appropriate (Gahm et al., 2012).

### Identifying Active Duty Army Decedents in NVDRS

We linked DoDSER data to NVDRS data to identify active duty U.S. Army decedents in NVDRS. Data linkage was initiated with DoDSER incident records. There were 154 active duty Army decedents who died in NVDRS states and therefore 154 DoDSER incident records; multivictim suicide incidents (e.g., suicide pacts) were not included. We linked the appropriate NVDRS record to each DoDSER record using a multistep process that compared common variables in both systems because all record were deidentified. For each DoDSER record, we first identified all NVDRS records that had the same manner, location (state), and date of death as well as decedent age and sex. Then, among the pool of selected NVDRS records, we selected the record that had the same decedent race/ethnicity and marital status, had documentation of the decedent “ever serving in the military,” and had documentation that the decedent had a current Army occupation (this documentation is provided as an open text field in NVDRS that lists terms like “soldier,” “Army Sgt”). If we selected an NVDRS record that did not match to its respective DoDSER record on all variables in the second step because of an unknown value, or because of a minor discrepancy, then we additionally reviewed the weapon information and the investigator narratives from both NVDRS and DoDSER records to ensure the stories of the suicide event captured by the two systems corroborated. Each DoDSER record only linked to one NVDRS record. In total, we linked NVDRS-DoDSER data for 141 (92%) Army suicide decedents. No personal identifying information was reviewed or appended.

### Identifying a Civilian Suicide Decedent Comparison Group in NVDRS

Each year, NVDRS collects details on over 10,000 civilian suicide incidents. To make a more manageable comparison between the Army versus civilian suicide decedents, we matched three to four NVDRS civilian suicide decedents to every Army decedent based on sex and age (within 1 year), state of death, and year of death to control for potential age, state, and sex specific social and environmental influences. We also considered matching by marital status and race/ethnicity, but small civilian case counts among some of the groups precluded ascertainment of three to four matches. Instead, marital status and race/ethnicity were used as control variables in the comparative analyses. Details on the process for matching civilian suicide decedents to Army decedents are provided in the online version of this article (please see link in the “Supporting Information” section). A total of 563 civilian suicide decedents were included.

### Variables

DoDSER data were used to describe the Army suicide decedent group with respect to component (Regular, Reserve, National Guard), duty status (active duty, activated Guard/Reserve, active duty for training), pay grade, recent military-related stresses, deployment history, and combat exposure.

NVDRS data were used to describe and compare the Army versus civilian suicide decedents with respect to demographic characteristics (age and sex [to verify matching], race/ethnicity, and marital status), place and method of death, as well as the precipitating factors as perceived by law enforcement agents, medical examiners, and their informants. The scope of decedent precipitating factors included the following: current/recent depressed mood or mental health problem; alcohol intoxication or suspected intoxication at the time of death; other substance abuse problems; a precipitating physical health problem (e.g., pain-related problem); a recent crisis (within 2 weeks of death); criminal or civil legal problems; job problems; financial problems; intimate partner problems; and/or other relationship problems. These factors have also been cited elsewhere as being known risk factors for suicide (Allen, Cross, & Swanner, 2005; Boscarino, 2006; Kung, Pearson, & Liu, 2003; Mahon, Tobin, Cusack, Kelleher, & Malone, 2005; Miller, Mogun, Azrael, Hempstead, & Solomon, 2008; Moscicki, 1995; Skopp et al., 2012; Thoresen & Mehlum, 2006). Additionally, we made comparisons based on other relevant preceding circumstances (e.g., prior suicide attempts, current mental health treatment, signs of premeditation). Definitions of all precipitating and other preceding circumstances of suicide in NVDRS are provided in Table1 (CDC, 2008).

**Table 1:** Precipitating and Other Preceding Circumstances of Suicide—National Violent Death Reporting System

**Precipitating circumstances**
* *Current depressed mood or mental health problem: decedent was perceived by self or others to be depressed or has been identified as having a mental health disorder or syndrome listed in the Diagnostic and Statistical Manual, version IV (DSM-IV)
* *Alcohol or other substance abuse problems or suspected intoxication: decedent was perceived by self or others to have a problem with, or to be addicted to, alcohol or other drugs. Or, the decedent was believed to be intoxicated at the time of death
* *Physical health problem: decedent was experiencing physical health problems that were believed to have contributed to the suicide (e.g., a recent cancer diagnosis or chronic pain)
* *Crisis during previous 2 weeks: a very current crisis or an acute precipitating event appeared to have contributed to the suicide. The crisis event must have occurred in the previous 2 weeks or be impending in the following 2 weeks (e.g., a trial for a criminal offense begins the following week)
* *Criminal or civil legal problems: decedent was facing criminal legal problems or civil legal problems (e.g., a child custody or civil lawsuit) that appeared to be associated with the suicide
* *Job problem: decedent was either experiencing a recent problem at work or was having a problem with joblessness
* *Financial problem: decedent was experiencing problems such as bankruptcy, overwhelming debt, or foreclosure of a home or business
* *Intimate partner problem: problems with a current or former intimate partner that appeared to have contributed to the suicide
* *Other relationship problem: problems with a family member, friend, or an associate (other than an intimate partner) that appeared to have contributed to the suicide.
**Other relevant preceding circumstances**
* *Current treatment for mental illness: decedent was currently receiving mental health treatment as evidenced by a current psychotropic medication or visited to a mental health professional in the previous 2 months
* *History of suicide attempts: decedent was known to have made previous attempts, regardless of the severity of those attempts
* *Person left a suicide note: decedent left a note, e-mail message, video, or other communication indicating an intent to die by suicide
* *Disclosed intent to die by suicide: decedent had previously expressed suicidal feelings to another person with time for that person to intervene; disclosure only at the time of the event, with no opportunity to intervene, is not coded as “disclosed intent to commit suicide”

### Analysis

We first described the military background of the U.S. Army decedents with the DoDSER data to obtain a better sense of this study group (please note that this study was unable to assess military-related risk factors). We then describe both the U.S. Army and the civilian suicide decedent groups with respect to demographic and incident characteristics as well the precipitating and preceding circumstances (i.e., provide the prevalence of each characteristics among both groups).

We compared the Army and civilian suicide decedent groups using conditional logistic regression. Simple conditional logistic regression models were used to make comparisons by demographic factors. Multivariable conditional logistic regression models were used to make comparisons with respect to location and method of death, the precipitating circumstances of death, and the other preceding circumstances. Matched prevalence odds ratios were used to assess associations. Odds ratios with additional adjustments for race/ethnicity and marital status were also provided. Comparisons made with respect to the precipitating circumstances accounted for all circumstance variables. Differences were considered significant at the 0.05 level.

## Results

### Military Characteristics of U.S. Army Suicide Decedents

Most Army decedents (86%) were in the active component (Regular) Army and 88% were in the enlisted ranks of E1 (Private) to E7 (Sergeant First Class) (Table2). A fifth of the Army decedents were identified as having recent military-related stresses. The most common stresses involved article 15 proceedings (nonjudicial proceedings for minor offenses administered by commanding officers), absent-without-leave proceedings, and/or courts-martial proceedings. An estimated 46% of Army decedents had at least one deployment and 19% had experienced combat. At least 19 soldiers were believed to have killed others, witnessed killing, or to have engaged in combat that resulted in casualties or wounded enemies.

**Table 2:** Military Background Characteristics of U.S. Army Suicide Decedents, 2005–2010^a^

Characteristic	No. (%) with Characteristic^b^ (*N* = 141)
Component	
Regular	121 (86)
Reserve	9 (6)
National Guard	11 (8)
Pay Grade	
E1–E2	14 (10)
E3	21 (15)
E4	40 (28)
E5	23 (16)
E6	14 (10)
E7	12 (9)
E8-E9	5 (4)
O1–10	8 (6)
W1–5 or other	4 (3)
Recent Military-Related Stresses	
Had any of the four stresses within 3 months of death:	28 (20)
Article 15, AWOL, and/or courts-martial proceedings	17 (12)
Administrative separation	11 (8)
Medical evaluation board	6 (4)
Not selected for promotion, schooling, or command	3 (2)
Deployment/Combat History	
Number of deployments:	
1–2	53 (38)
3 or more	11 (8)
Unknown	26 (18)
Known to have orders to deploy	10 (7)
Experienced direct combat	27 (19)
Within the last three deployments:	
Engaged in combat resulting in wounded	19 (13)
Sustained an injury resulting from combat	5 (4)
Witnessed killing	15 (11)
Killed others	13 (9)

*Note*. E, enlisted ranks; W, warrant officer ranks; O, commissioned officer ranks; AWOL, absent-without-leave.

a Data were provided from Department of Defense Suicide Event Report (DoDSER) and the Army Suicide Event Report.

b Percentages for each variable might not equal 100% because of rounding.

### Demographic Characteristics

The demographic and incident characteristics of the decedents are shown in Table3. The Army and civilian decedent groups were matched by age and sex, and therefore, the two groups did not differ by these characteristics as expected. The majority (88%) of all decedents were aged 18 to 39 years (because of the matching criteria, some civilian decedents were 17 years of age). Most decedents (96%) were males. Also, both groups did not significantly differ with respect to race/ethnicity. Most Army decedents (72%) and civilian decedents (76%) were of non-Hispanic White race/ethnicity.

**Table 3:** Demographic and Incident Characteristics for US Army versus Civilian Suicide Decedents, 2005–2010^a^

Characteristic	No. (%) with Characteristic		Matched Prevalence Odds Ratios, 95% Confidence Intervals^b^	
	US Army Suicide Decedents (*n* = 141)	Civilian Suicide Decedents (*n* = 563)	Crude	Adjusted for Race, Ethnicity, and Marital Status
Age (years)				
17–24	61 (43)	240 (43)	n/a	n/a
25–29	34 (24)	139 (25)	n/a	n/a
30–39	30 (21)	120 (21)	n/a	n/a
40–59	16 (11)	64 (11)	n/a	n/a
Mean (standard deviation)	28.0 (8.0)	28.0 (8.2)		
Sex				
Male	135 (96)	539 (96)	n/a	n/a
Female	6 (4)	24 (4)	n/a	n/a
Race/ethnicity				
White, non-Hispanic	101 (72)	430 (76)	**	n/a
Black, non-Hispanic	19 (13)	67 (12)	1.20 (0.67–2.12)	n/a
Hispanic	11 (8)	34 (6)	1.42 (0.67–2.99)	n/a
Other	10 (7)	28 (5)	1.61 (0.72–3.60)	n/a
Marital status				
Never married	47 (33)	360 (64)	**	
Married	82 (58)	124 (22)	7.75 (4.65–12.92)	n/a
Widowed, divorced, or separated	10 (7)	63 (11)	1.84 (0.83–4.10)	n/a
Location of death				
Residential area	98 (70)	423 (75)	**	**
Transport area (public roads, inside vehicle)	13 (9)	48 (9)	1.23 (0.65–2.34)	1.00 (0.49–2.01)
Recreational, commercial, or natural areas	14 (10)	69 (12)	0.87 (0.47–1.63)	0.89 (0.45–1.77)
Other^c^	12 (9)	20 (4)	3.18 (1.38–7.33)	3.23 (1.23–8.46)
Mechanism				
Firearm	89 (63)	317 (56)	**	**
Poisoning	13 (9)	41 (7)	1.11 (0.56–2.21)	1.18 (0.54–2.60)
Hanging, strangulation	25 (18)	165 (29)	0.51 (0.31–0.83)	0.50 (0.30–0.86)
Other	8 (6)	36 (6)	0.71 (0.31–1.62)	0.65 (0.26–1.64)

*Note*. n/a, not applicable.

a Data were provided by the National Violent Death Reporting System. Unknown values are not presented; therefore, variables might not total 100%.

b All odds ratios accounted for all variables in the match (i.e., state of injury, year of death, and age and sex of decedent). Civilian suicide decedents were the referent population.

** Identifies the referent level for each variable. For each variable, Army decedents with unknown values and their civilian matches were excluded from the comparison; no more than 5% of the groups were excluded because of unknown values. The odds ratios = odds of the exposure among Army decedents divided by the odds of the exposure among civilian decedents.

c Other specified locations of death included schools and sports/athletic areas.

In contrast, there were clear differences between the two groups with respect to marital status. Over half (58%) of the Army decedents versus only 22% of the civilian decedents were married (*p* < .05). Furthermore, 64% of the civilian decedents versus 33% of Army decedents were never married (*p* < .05).

### Incident Characteristics

The majority of Army (70%) and civilian (75%) decedents died in personal residences, followed by recreational/commercial/natural areas (10% Army and 12% civilian), and transport areas (9% of each group). A higher proportion of Army versus civilian decedents died in other locations. The most common mechanism of death for both groups was firearm use (63% Army and 56% civilian). Hanging was the second most common mechanism for both groups (18% Army and 29% civilian), but this mechanism was more prevalent among civilian decedents (*p* < .05).

### Precipitating and Other Preceding Circumstances

We did not find any significant between-group differences with respect to precipitating health- and stress-related circumstances based on the adjusted models. The five most common precipitators of suicide for all decedents, based on the investigator reports, were having mental health-related problems (53% Army and 64% civilian), intimate partner problems (54% Army and 42% civilian), alcohol or substance abuse problems (41% Army and 48% civilian), recent crises (37% Army and 33% civilian), and job problems (18% Army and 16% civilian) (see Table4). Also, an estimated 27% of both decedent groups left suicide notes, which often indicates premeditation.

**Table 4:** Precipitating and Other Preceding Circumstances of Death for U.S. Army versus Civilian Suicide Decedents, 2005–2010^a^

Characteristic	Level	No. (%) with Characteristic		Matched Prevalence Odds Ratios, 95% Confidence Intervals^b^		
		U.S. Army Suicide Decedents (*n* = 104)	Civilian Suicide Decedents (*n* = 416)	Crude	Adjusted for Race, Ethnicity, and Marital Status	Adjusted for All Demographic and Recent Circumstance Variables^c^
Precipitating Health Circumstances						
Current depressed mood or mental health problem	No/unknown	49 (47)	150 (36)	**	**	**
	Yes	55 (53)	266 (64)	0.62 (0.40–0.97)	0.73 (0.43–1.23)	0.70 (0.41–1.21)
Alcohol dependence, other substance abuse, or suspected intoxication	No/unknown	61 (58)	215 (52)	**	**	**
	Yes	43 (41)	201 (48)	0.76 (0.49–1.17)	0.69 (0.42–1.13)	0.68 (0.41–1.12)
Physical health problems	No/unknown	95 (91)	388 (93)	**	**	**
	Yes	9 (9)	28 (7)	1.36 (0.59–3.15)	1.19 (0.44–3.27)	1.23 (0.45–3.37)
Precipitating Stress-Related Circumstances						
Recent crisis (within past 2 weeks)	No/unknown	65 (63)	280 (67)	**	**	**
	Yes	39 (37)	136 (33)	1.27 (0.79–2.03)	1.09 (0.63–1.89)	1.04 (0.55–1.97)
Criminal and civil legal problems	No/unknown	89 (86)	349 (84)	**	**	**
	Yes	15 (14)	67 (16)	0.88 (0.48–1.61)	0.73 (0.36–1.46)	0.67 (0.32–1.42)
Financial problems	No/unknown	92 (88)	375 (90)	**	**	**
	Yes	12 (12)	41 (10)	1.19 (0.60–2.36)	1.30 (0.61–2.77)	1.32 (0.60–2.89)
Job problems	No/unknown	85 (82)	349 (84)	**	**	**
	Yes	19 (18)	67 (16)	1.16 (0.67–2.03)	0.95 (0.50–1.82)	1.00 (0.51–1.98)
Intimate partner problems	No/unknown	48 (46)	243 (58)	**	**	**
	Yes	56 (54)	173 (42)	1.62 (1.06–2.49)	0.91 (0.55–1.51)	0.97 (0.56–1.70)
Other relationship problems	No/unknown	91 (87)	368 (88)	**	**	**
	Yes	13 (13)	48 (12)	1.10 (0.57–2.11)	1.70 (0.83–3.51)	1.86 (0.86–4.02)
Other Relevant Preceding Circumstances						
Recent mental health treatment	No/unknown	76 (73)	298 (72)	**	**	**
	Yes	28 (27)	118 (28)	0.93 (0.57–1.51)	1.16 (0.66–2.01)	1.19 (0.66–2.13)^d^
History of suicide attempts	No/unknown	96 (92)	332 (80)	**	**	**
	Yes	8 (8)	84 (20)	0.32 (0.15–0.68)	0.28 (0.12–0.65)	0.29 (0.12–0.68)^d^
Premeditation						
Left a note	No/unknown	76 (73)	304 (73)	**	**	**
	Yes	28 (27)	112 (27)	1.00 (0.61–1.64)	0.88 (0.50–1.55)	0.95 (0.53–1.70)
Disclosed intent	No/unknown	84 (81)	290 (70)	**	**	**
	Yes	20 (19)	126 (30)	0.54 (0.32–0.93)	0.53 (0.29–0.96)	0.52 (0.28–0.97)

a Data were provided by the National Violent Death Reporting System. Estimates were calculated for Army cases with *known* law enforcement and/or medical examiner circumstance information and their respective matched civilian cases (Army cases = 104; civilian cases = 416).

b All matched prevalence odds ratio estimates accounted for the variables in the match (i.e., state of injury, year of death, and age and sex of decedent). Civilian suicide decedents were the referent population.

** Identifies the referent level for each variable. The odds ratios = odds of the exposure among Army decedents divided by the odds of the exposure among civilian decedents.

c These estimates also adjusted for race/ethnicity, marital status, and all other recent health and stress-related circumstance variables aside from the circumstance of interest. Recent circumstance variables included current depressed mood or mental health problems, alcohol of other substance abuse problems or suspected intoxication, physical health problems, recent crisis, legal problems, financial problems, job problems, intimate partner problems, and other relationship problems.

d To prevent collinearity, the variable “current depressed mood or mental health problems” was excluded from the regression model.

We initially found that a higher proportion of Army versus civilian decedents had precipitating intimate partner problems based on the crude matched odds ratio of 1.62 (*p* < .05); however, this difference was not significant after accounting for both race/ethnicity and marital status as well as marital status alone (data not shown). Furthermore, there were no differences in the prevalence of intimate partner problems between the two decedent groups in each marital status category (data not shown).

A smaller proportion of Army versus civilian decedents was also initially found to have a known current depressed mood and/or mental health problem; however, this difference was not significant after accounting for race/ethnicity and marital status. Furthermore, we did not find significant differences between the groups with respect to the proportion of decedents who were currently receiving mental health treatment; an estimated 27% of Army and 28% of civilian decedents were receiving mental health treatment near the time of death.

With respect to prior suicidal ideation and behavior, we found a smaller proportion of Army versus civilian decedents had prior suicidal attempts and a smaller proportion of Army versus civilian decedents disclosed suicidal intentions to others (Table4). These differences remained significant across all adjusted regression models (all odds ratios were significant at the 0.05 level).

## Discussion

This study showed that the precipitating factors of suicide as perceived by law enforcement and medical examiner investigators, family members, friends, other acquaintances of the victims, and other informants, overall, were not significantly different between Army and civilian suicide decedents. These suicides were commonly believed to be triggered by mental health and/or intimate partner problems. Substance misuse/abuse, recent crises, job, legal, and financial problems were also believed to have played a role in many of these deaths.

Intimate partner problems were the most common precipitating factors involved in suicide among Army decedents and the most common stress-related precipitating factors of suicide among civilian decedents according to the investigator reports. Other studies have shown that male versus female suicide decedents more commonly have intimate partner problems preceding death (Karch, Logan, McDaniel, Parks, & Patel, 2012; Karch, Logan, & Patel, 2011); therefore, a high prevalence of such problems was anticipated for these predominantly male suicide groups. Furthermore, the link between suicide and relationship problems has been found among military personnel (Hyman et al., 2012; Skopp et al., 2012) as well as in civilian populations (Logan et al., 2011; Moscicki, 1995). The civilian literature has indicated that positive healthy intimate partner relationships may serve a protective function against suicide (Aro, 1994; California Healthy Marriages Coalition, 2009; Smith, Mercy, & Conn, 1988). Given the high percentage of married enlisted soldiers in the Army (41% among rank E1–E4 and 73% among ranks E5–E6; Department of Defense, 2010), identifying strategies that improve intimate partner relationships may benefit military suicide prevention efforts.

Mental health problems were also believed to be contributing factors in over half of Army suicides, a finding that was also similar among civilian suicide decedents. Mental health problems are not only associated with increased risk of suicide (Bachynski et al., 2012; Hyman et al., 2012; Skopp et al., 2012), but also with increased risk of spousal discord (Gibbs, Clinton-Sherrod, & Johnson, 2012). This study also found that over 25% of Army and civilian decedents were receiving mental health treatment near the time of death, which supports evidence that more comprehensive strategies intended to prevent self-directed violence—beyond mental health treatment alone—are needed to help prevent suicide (Logan et al., 2011).

Army suicide decedents less commonly disclosed suicidal intentions than civilian decedents, and a higher proportion of Army versus civilian decedents did not have prior attempts. These findings might be attributable to a couple of factors unique to the Army. The Army may have more timely or systematic interventions in place to respond to those who disclose suicide intent or have nonfatal attempts than the general civilian population; therefore, they have a smaller proportion of decedents with prior suicidal ideation and behavior because they are better at preventing subsequent attempts. Or, Army suicide decedents may be less likely than civilian suicide decedents to disclose suicide intentions or have prior nonfatal attempts. In either case, there appears to be a need for additional prevention efforts in the Army for soldiers who do not disclose suicidal intent to achieve further reductions in suicidal mortality. Soldiers need to be aware of suicidal warning signs and reach out to persons showing such signs to connect them to needed sources of support. Such a strategy is consistent with the Army's current approach to suicide prevention, which encourages soldiers to support each other and to be aware of signs of distress (Ask, Care, Escort [ACE] program; details of the Army ACE Suicide Intervention Program can be found at http://phc.amedd.army.mil/topics/healthyliving/bh/Pages/SuicidePreventionEducation.aspx).

Several limitations of this study should be considered. First, this study only included U.S. states participating in NVDRS, and therefore, our findings are not nationally representative. Second, this study did not assess risk factors but only factors that were believed to have been involved in the deaths based on death scene investigator reports, the witnesses, and the material evidence collected. These details are limited to the knowledge of the informants and the thoroughness of the investigations and the documentation; however, law enforcement and coroner/medical examiner death scene investigators are held to a standard to ensure these deaths were not the result of criminal activity. Third, mental health information was not often captured from medical records but from coroner/medical examiner reports, family members, and friends of the decedents; therefore, the completeness of this information was limited based on the knowledge of the informant.

Data systems available through the CDC and DoD provide valuable insights to help understand suicide in military populations. These data systems can be integrated even when deidentification is required and provide complementary information that can be of value. However, even these systems together do not fully answer the complex questions of why military suicide rates are increasing and what should be done to change this trajectory. Further research is clearly needed to determine how these factors may be interrelated and what actions should be recommended to make changes. Clearly, data sets such as those of NVDRS and the DoDSER are critical to ongoing surveillance of this issue and supporting the ability to objectively evaluate any interventions that are instituted.

## Supporting Information

Appendix S1 Process for matching civilian suicide decedents to U.S. army suicide decedents in NVDRS.

## References

[b1] Allen JP, Cross G, Swanner J (2005). Suicide in the Army: A review of current information. Military Medicine.

[b2] Aro H (1994). Risk and protective factors in depression: A developmental perspective. Acta Psychiatrica Scandinavica.

[b3] Bachynski KE, Canham-Chervak M, Black SA, Dada EO, Millikan AM, Jones BH (2012). Mental health risk factors for suicides in the U.S. Army, 2007–2008. Injury Prevention.

[b4] Boscarino JA (2006). Posttraumatic stress disorder and mortality among U.S. Army veterans 30 years after military service. Annals of Epidemiology.

[b5] California Healthy Marriages Coalition (2009). http://www.camarriage.com/content/resources/c18f014d-51a5-43e4-ba68-e032fbcc22ee.pdf.

[b6] Centers for Disease Control and Prevention (2008). http://www.cdc.gov/violenceprevention/pdf/NVDRS_Coding_Manual_Version_3-a.pdf.

[b7] Department of Defense, Office of the Deputy Under Secretary of Defense (2010). http://www.ncdsv.org/images/DOD_DemographicsProfileOfTheMilitaryCommunity_2010.pdf.

[b8] Department of Defense (2012). http://www.health.mil/dhb/downloads/Suicide%20Prevention%20Task%20Force%20final%20report%208-23-10.pdf.

[b9] Eaton KM, Messer SC, Garvey Wilson AL, Hoge CW (2006). Strengthening the validity of population-based suicide rate comparisons: An illustration using U.S. military and civilian data. Suicide and Life-Threatening Behavior.

[b10] Gahm GA, Reger MA, Kinn JT, Luxton DD, Skopp NA, Bush NE (2012). Addressing the surveillance goal in the National Strategy for Suicide Prevention: The Department of Defense suicide event report. American Journal of Public Health.

[b11] Gibbs DA, Clinton-Sherrod AM, Johnson RE (2012). Interpersonal conflict and referrals to counseling among married soldiers following return from deployment. Military Medicine.

[b12] Helmkamp JC (1995). Suicides in the military: 1980–1992. Military Medicine.

[b13] Hyman J, Ireland R, Frost L, Cottrell L (2012). Suicide incidence and risk factors in an active duty U.S. military population. American Journal of Public Health.

[b14] Kang HK, Bullman TA (2008). Risk of suicide among U.S. veterans after returning from the Iraq or Afghanistan war zones. Journal of the American Medical Association.

[b15] Karch DL, Logan JE, McDaniel D, Parks S, Patel N (2012). Surveillance for violent deaths— National Violent Death Reporting System, 16 states, 2009. Morbidity and Mortality Weekly Report Surveillance Summary.

[b16] Karch DL, Logan J, Patel N (2011). Surveillance for violent deaths— National Violent Death Reporting System, 16 states, 2008. Morbidity and Mortality Weekly Report Surveillance Summary.

[b17] Kuehn BM (2009). Soldier suicide rates continue to rise: Military, scientists work to stem the tide. Journal of the American Medical Association.

[b18] Kung HC, Pearson JL, Liu X (2003). Risk factors for male and female suicide decedents ages 15–64 in the United States: Results from the 1993 national mortality followback survey. Social Psychiatry and Psychiatric Epidemiology.

[b19] Lineberry TW, O'Connor SS (2012). Suicide in the U.S. Army. Mayo Clinic Proceedings.

[b20] Logan J, Hall J, Karch D (2011). Suicide categories by patterns of known risk factors: A latent class analysis. Archives of General Psychiatry.

[b21] Logan J, Hill HA, Black ML, Crosby AE, Karch DL, Barnes JD (2008). Characteristics of perpetrators in homicide-followed-by-suicide incidents: National Violent Death Reporting System–17 U.S. states, 2003–2005. American Journal of Epidemiology.

[b22] Logan J, Skopp NA, Karch D, Reger MA, Gahm GA (2012). Characteristics of suicides among U.S. army active duty personnel in 17 U.S. states from 2005 to 2007. American Journal of Public Health.

[b23] Luxton DD, Osenbach JE, Reger MA, Smolenski DJ, Skopp NA, Bush NE (2012).

[b24] Mahon MJ, Tobin JP, Cusack DA, Kelleher C, Malone KM (2005). Suicide among regular-duty military personnel: A retrospective case-control study of occupation-specific risk factors for workplace suicide. American Journal of Psychiatry.

[b25] Miller M, Mogun H, Azrael D, Hempstead K, Solomon DH (2008). Cancer and the risk of suicide in older Americans. Journal of Clinical Oncology.

[b26] Moscicki EK (1995). Epidemiology of suicide. International Psychogeriatrics.

[b27] Skopp NA, Trofimovich L, Grimes J, Oetjen-Gerdes L, Gahm GA (2012). Relations between suicide and traumatic brain injury, psychiatric diagnoses, and relationship problems, active component, U.S. Armed Forces, 2001–2009. Medical Surveillance Monthly Report.

[b28] Smith JC, Mercy JA, Conn JM (1988). Marital status and the risk of suicide. American Journal of Public Health.

[b29] Thoresen S, Mehlum L (2006). Suicide in peacekeepers: Risk factors for suicide versus accidental death. Suicide and Life-Threatening Behavior.

[b31] U.S. Department of Justice, Office of Justice Programs, National Institute of Justice (1999). https://www.ncjrs.gov/pdffiles/167568.pdf.

[b32] U.S. Surgeon General and the National Action Alliance for Suicide Prevention (2012). http://www.surgeongeneral.gov/library/reports/national-strategy-suicide-prevention/full-report.pdf.

