# Supplementary material for: Precipitating Circumstances of Suicide among Active Duty U.S. Army Personnel Versus U.S. Civilians, 2005–2010
Source: Suicide Life Threat Behav. 2014 Aug 5;45(1):65–77. doi: 10.1111/sltb.12111 (PMC4384804; doi:10.1111/sltb.12111)
Supplement: Appendix S1 — Process for matching civilian suicide decedents to U.S. army suicide decedents in NVDRS. [file sltb0045-0065-sd1.docx]

**APPENDIX OR ONLINE-ONLY MATERIAL**

**Process for Matching Civilian Suicide Decedents to US Army Suicide Decedents in NVDRS**

Selection for NVDRS civilian suicide decedents was initiated with those who: 1) had known circumstance information from law enforcement and medical examiner records; 2) were involved in single suicide incidents, not homicide-followed-by-suicide incidents or multiple suicide incidents; and 3) died within the years of 2005-2010. An estimated 51,970 (84.6%) of 61,395 NVDRS suicide decedents met these criteria. The Army suicide incidents were assigned random numbers and then sorted. Starting with the first Army incident, we matched all civilian suicide incidents based on year of occurrence, state of injury/death, decedent sex, and decedent age (plus or minus 1 year). If more than four civilian suicide incidents matched, then four were randomly selected. Once selected, we reviewed text fields that listed the civilian decedents’ occupations to ensure we did not select military service members. Civilian suicide decedents that were finally selected were removed from the pool of potential matches before the selection process was repeated for the next Army incident.
